# The role of pudendal nerve block in hemorrhoid surgery: a systematic review and meta-analysis of double-blind randomized controlled trials

**DOI:** 10.3389/fmed.2023.1283512

**Published:** 2023-12-13

**Authors:** Shijun Xia, Lidan Luo, Wenjiang Wu, Kaiyuan Lu, Tao Jiang, Yue Li

**Affiliations:** Shenzhen Hospital of Guangzhou University of Chinese Medicine, Shenzhen, China

**Keywords:** hemorrhoid, surgery, pudendal nerve block, anesthesia, visual analog scale

## Abstract

**Background:**

Pudendal nerve block (PNB) is a commonly used anesthesia method that has been widely used in postoperative analgesia for hemorrhoids in recent years. Therefore, we conducted a systematic review and meta-analysis of double-blind randomized controlled trials (RCTs) to analyze the effectiveness of PNB in postoperative analgesia for hemorrhoids.

**Methods:**

Relevant data and studies published from inception until August 14, 2023, were retrieved from PubMed, Embase, and Web of Science to evaluate the beneficial effects of PNB for analgesia following hemorrhoidectomy.

**Results:**

This meta-analysis included 6 double-blind RCTs comprising 501 patients. We evaluated the function of PNB in improving outcomes of postoperative analgesia of hemorrhoids. Visual analogue scale (VAS) scores on postoperative within 6 h (MD, −3.04; 95% CI, −4.13 to −1.95; *P* < 0.0001), 12 h (MD, −3.14; 95% CI, −3.87 to −2.40; *P* < 0.0001), and 24 h (MD, −2.25; 95% CI, −2.95 to −1.55; *P* < 0.0001) were enhanced by the application of PNB, but not in 48 h (MD, −2.54; 95% CI, −5.29 to 0.20; *P* = 0.07).

**Conclusion:**

Pudendal nerve block (PNB) could effectively relieve postoperative pain of hemorrhoids. However, our results still need to be confirmed by multi-center clinical studies.

## 1 Introduction

Hemorrhoids are normal vascular structures in the anal canal. Approximately 5% of the general population suffers from hemorrhoidal disease (1), the cardinal features of which include bleeding, anal pruritus, prolapse, and pain due to thrombosis or inflammatory crisis. Most patients with hemorrhoidal disease initially undergo conservative treatment. In cases where internal medicine treatment is unsuccessful, non-surgical outpatient treatment procedures may be suitable. Only patients with persistent symptoms after conservative or outpatient treatment are considered candidates for surgical intervention.

Pain after hemorrhoidectomy is extremely common and is caused, to some extent, by spasms of the anal sphincter (2). The first-choice medication for controlling postoperative pain includes oral analgesics, such as non-steroidal anti-inflammatory drugs and acetaminophen (3). When these analgesics are ineffective for pain control, opioid drugs can be used; however, they may cause adverse reactions such as constipation. The PROSPECT group recommends the application of a pudendal nerve block (PNB) to relieve postoperative pain in patients who have undergone hemorrhoidectomy (4).

The pudendal nerve is a mixed sensory and motor nerve originating from the S2, S3, and S4 nerve roots of the sacral plexus (5). It exits the pelvis through the greater sciatic foramen and then re-enters the perineum, passing through the ischiorectal fossa and Alcock’s canal. Here, the pudendal nerve is accompanied by pudendal blood vessels and splits into three branches. The pudendal nerve innervates the urethral muscles, clitoris, penis, perineum, pelvic floor sphincters, urethra, and bladder triangle (6, 7).

Pudendal nerve blocks (PNBs) are suitable for postoperative pain relief following hemorrhoidectomy and are considered an optional pain management strategy. Multiple randomized trials have been published on PNBs, confirming a longer analgesic duration and lower incidence of complications compared with the abovementioned drugs (8, 9). Although some systematic reviews and meta-analyses support the analgesic effect of PNBs in hemorrhoid surgery (10, 11), most of them are based on low-quality research data.

Therefore, we expanded the search scope and performed an updated meta-analysis based on all published double-blind randomized controlled trials to evaluate the beneficial effects of PNBs for analgesia following hemorrhoidectomy.

## 2 Materials and methods

This systematic review and meta-analysis was performed in accordance with the Preferred Reporting Items for Systematic Reviews and Meta-Analyses guidelines (12). Ethical approval or informed consent was not required.

### 2.1 Search strategy

We searched the PubMed, Embase, and Web of Science electronic databases for English articles from database inception until August 14, 2023. The following search terms were used: (“pudend*” or “ischiorectal”) and (“anesthesia” or “anaesthesia”), and (“hemorrhoids” or “haemorrhoid” or “hemorrhoidectomy” or “haemorrhoidectomy”). The search strategy was implemented using a combination of index words and free-text keywords.

### 2.2 Eligibility criteria

Two researchers (LL and KL) analyzed and independently reviewed all studies retrieved through the literature search. Any disagreements were resolved through discussion. The inclusion criteria were as follows: (1) double-blind randomized controlled trials, (2) investigation of patients with hemorrhoidal disease, (3) comparison of PNBs with any other treatment, and (4) studies published in English. The exclusion criteria were as follows: (1) studies involving participants with non-hemorrhoidal disease and (2) those involving perianal blocks. Following the application of the inclusion and exclusion criteria, a full-text evaluation of the remaining potentially eligible studies was conducted to determine whether they should be included in the analysis. Additionally, the reference lists of the evaluated full-text articles were reviewed to make the search more comprehensive.

### 2.3 Data collection and outcomes

Data for each eligible study were extracted, including the title, author, year, country, grouping method, number of patients, randomization type, blinding method, PNB technique, and postoperative pain data. The primary endpoint pain was postoperative pain. This was measured using a visual analog scale (VAS), which scored perceived pain on a level of 0–10 during rest, walking, sitting, or the first bowel movement.

### 2.4 Quality assessment

An independent review of bias risk and research quality was conducted based on the Cochrane Collaboration tool for assessing risk of bias (13).

### 2.5 Statistical methods

Review Manager v5.3 (Nordic Cochrane Center, Cochrane Collaboration, London, UK) was used for data analysis. We calculated the intergroup standardized mean difference (SMD) for each study as an estimate of effectiveness. In case of missing data, the mean and standard deviation (SD) were estimated based on the median, range, and interquartile values (14, 15). *I*^2^ values were used to classify heterogeneity as follows: no heterogeneity, *I*^2^ < 25%; low heterogeneity, 25% ≤ *I*^2^ < 50%; moderate heterogeneity, 50% ≤ *I*^2^ < 75%; and high heterogeneity, *I*^2^ ≥ 75%. When the *I*^2^ value was < 50%, the fixed effects model was used, whereas the random effects model was used when the *I*^2^ value was > 50%.

## 3 Results

### 3.1 Selected studies

**FIGURE 1:**
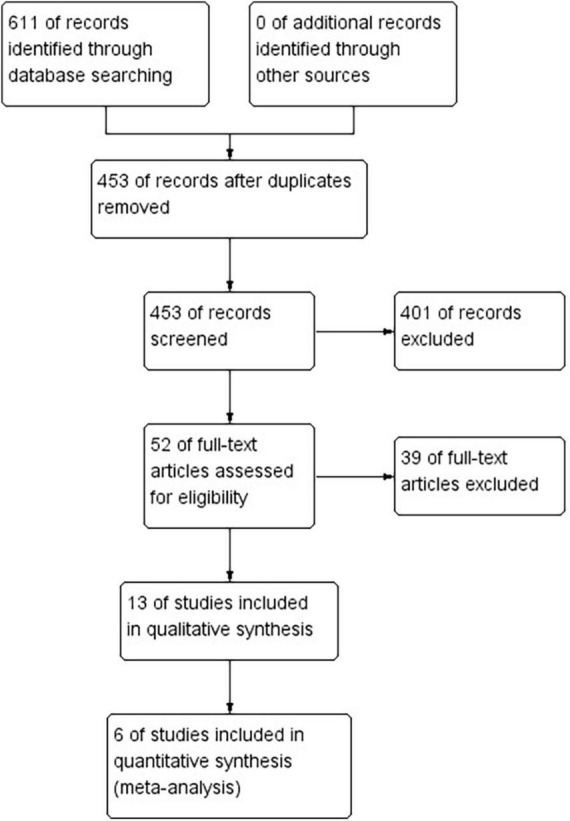
PRISMA flow diagram of study selection.

Figure 1 describes the process of literature retrieval and screening. A total of 611 studies were retrieved, of which 453 were evaluated after eliminating duplications. Further, 401 studies were excluded because they did not meet the inclusion criteria, and only 6 (16–21) of the remaining 13 potential studies were ultimately included in this study. These 6 studies were published between 2000 and 2020, and included 501 patients from 6 countries, with a sample size of 20–200. The PNB procedure was achieved in one study under ultrasound guidance, in two studies under nerve stimulation and in the other three under anatomic landmarks (Table 1).

**TABLE 1:** Characteristics of the included trials.

References	Country	Type of groups	Number of participants	Randomization and blinding	PNB technique	Observation time of pain	Quality assessment
Di Giuseppe et al. (21)	Switzerland	SP + PNB	23	1:1 ratio; double-blind	Ultrasound guided	6, 12, 24, and 48 h	Good
		SP	26				
Rajabi et al. (20)	Iran	GA + PNB	30	1:1:1 ratio; double-blind	Anatomic landmarks	12 h	Good
		GA	30				
		GA + placebo PNB	30				
Imbelloni et al. (19)	>Brazil	SP + PNB	100	>1:1 ratio; double-blind	>Under nerve stimulation	>6, 12, 18, 24, and 30 h	>Good
		SP	100				
Naja et al. (18)	Lebanese	GA + PNB	30	1:1:1 ratio; double-blind	Under nerve stimulation	6 h, 12 h, 24 h, 36 h, 48 h, 3 days, 4 days, 5 days, and 6 days	Good
		GA	30				
		GA + placebo PNB	30				
Brunat et al. (17)	France	GA + PNB	25	1:1 ratio; double-blind	Anatomic landmarks	1, 2, 4, 8, 12, and 24 h	Good
		GA	27				
Luck and Hewett (16)	Australia	GA + PNB + LA	10	1:1 ratio; double-blind	Anatomic landmarks	0.5, 2, 4, and 24 h	Good
		GA + LA	10				

### 3.2 Quality assessment

**FIGURE 2:**
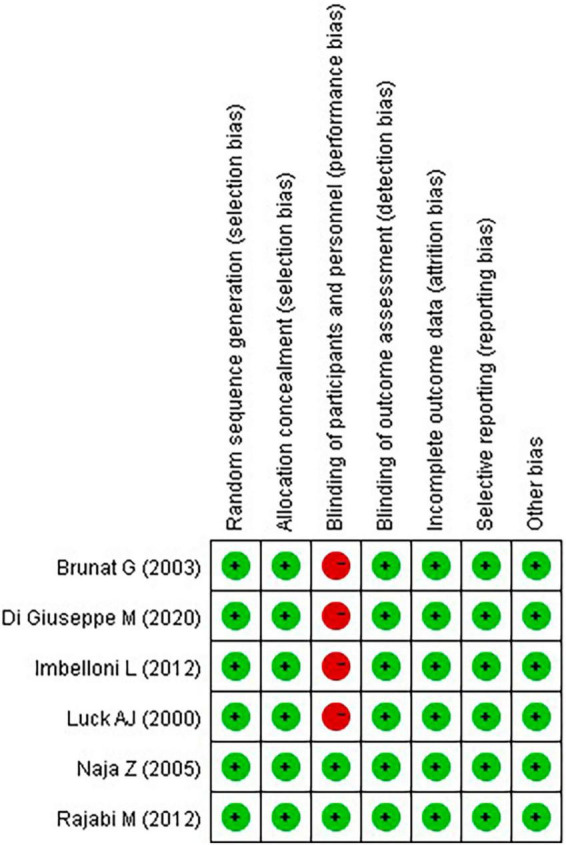
Summary of the risk of bias.

**FIGURE 3:**
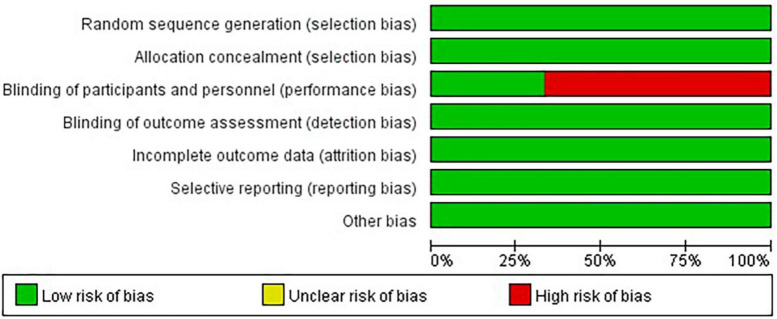
Graph of the risk of bias.

Figures 2, 3 present the results of the Cochrane risk of bias tool. Considering the nature of the intervention, all studies were not conducted blind analysis of researchers.

### 3.3 Outcomes

For each study, we compared the VAS scores at different time points between the experimental and control groups to evaluate the differences in analgesic effects between the two groups.

### 3.4 Pain within 6 h on the VAS

**FIGURE 4:**
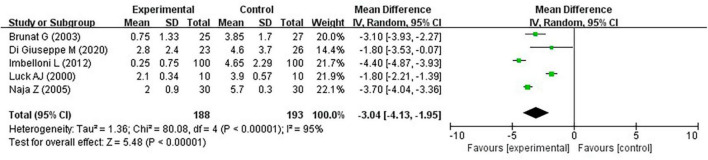
Pain within 6 h on the VAS.

According to the studies by Brunat et al. (17), Di Giuseppe et al. (21), Imbelloni et al. (19), Luck and Hewett (16), and Naja et al. (18), VAS scores within 6 h of hemorrhoidectomy were lower in the experimental group than in the control group (MD, −3.04; 95% confidence interval [CI], −4.13 to −1.95; *P* < 0.0001; *I*^2^ = 95%) (Figure 4).

### 3.5 Pain at 12 h on the VAS

**FIGURE 5:**
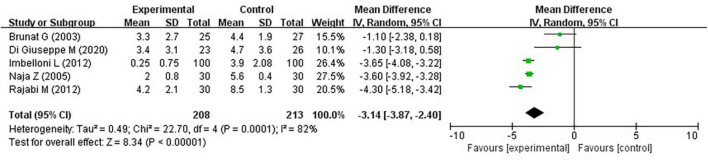
Pain at 12 h on the VAS.

Brunat et al. (17), Di Giuseppe et al. (21), Imbelloni et al. (19), Naja et al. (18), and Rajabi et al. (20) reported that the VAS score at 12 h after hemorrhoidectomy was lower in the experimental group than in the control group (MD, −3.14; 95% CI, −3.87 to −2.40; *P* < 0.0001; *I*^2^ = 82%) (Figure 5).

### 3.6 Pain at 24 h on the VAS

**FIGURE 6:**
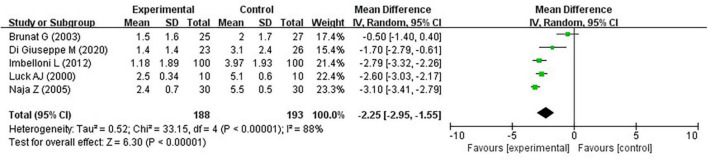
Pain at 24 h on the VAS.

Brunat et al. (17), Di Giuseppe et al. (21), Imbelloni et al. (19), Luck and Hewett (16), and Naja et al. (18), reported that the VAS score at 24 h after hemorrhoidectomy was also lower in the experimental group (MD, −2.25; 95% CI, −2.95 to −1.55; *P* < 0.0001; *I*^2^ = 88%) (Figure 6).

### 3.7 Pain at 48 h on the VAS

**FIGURE 7:**
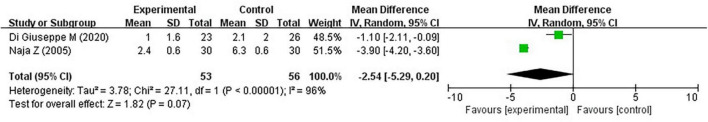
Pain at 48 h on the VAS.

However, at 48 h after hemorrhoidectomy, Di Giuseppe et al. (21) and Naja et al. (18) reported no difference in VAS scores between the experimental and control groups (MD, −2.54; 95% CI, −5.29 to 0.20; *P* = 0.07; *I*^2^ = 96%) (Figure 7).

### 3.8 Publication bias

As the number of included studies was < 10, we could not evaluate publication bias.

## 4 Discussion

Our systematic review and meta-analysis based on six high-quality double-blind randomized controlled trials revealed that the use of PNBs in patients undergoing hemorrhoidectomy can reduce pain within 6, 12, and 24 h after surgery, but it is ineffective 48 h after surgery. Thus, our findings support the wider use of PNBs in hemorrhoidectomy.

Hemorrhoids are detected in many anorectal diseases. Although surgery is the most effective treatment method, postoperative pain is one of the main reasons attributed to surgery refusal (22). The main sources of post-hemorrhoidectomy pain are the surgical incision site, perianal skin, and areas of mucosal edema (23). Because the perineum is extremely sensitive, patients undergoing hemorrhoidectomy always experience severe postoperative pain. Perioperative analgesia mainly relies on local anesthesia and painkillers (24) as well as local application of diltiazem (25) and injections of botulinum toxin (26) or metronidazole (topical or oral) (27, 28). Tail or spinal anesthesia can also relieve pain, but the analgesic effect is short-lived, often accompanied with side effects, particularly urinary retention (29). Local infiltration can alleviate postoperative pain in patients undergoing hemorrhoid surgery, but this pain relief only lasts for 5–12 h (30, 31). Further, improvement in their analgesic effects remains unsatisfactory.

Pudendal nerve blocks (PNBs) are mainly used for anorectal diseases, and they alleviate pain by blocking the anal nerve at the bifurcation of the pubic nerve. Due to its anatomical structure, PNBs can be an effective post-hemorrhoidectomy analgesic method. Complications related to PNB surgery, such as intravenous anesthesia, permanent nerve injury, hematoma, and abscess, have never been described in relevant literature searches, although there may be reporting bias. A meta-analysis not only confirmed the highly beneficial safety of PNBs but also showed that the incidence of nausea and vomiting were significantly reduced (10). PNBs were also reported to reduce the incidence of urinary retention (11), which is a relatively common complication after hemorrhoid surgery that may hinder outpatient treatment.

This study has some limitations. The most relevant limitation is related to publication bias and heterogeneity, with high *I*^2^ values in various studies. Furthermore, the sample size included in some studies was relatively small, which may cause bias. Finally, in cases of missing data, the impact was estimated using mean and SD, which may not accurately reflect the original data.

## 5 Conclusion

Our meta-analysis and systematic review of the studies extracted from the literature revealed that the use of PNBs in patients undergoing hemorrhoidectomy has a significant advantage in alleviating postoperative pain. Despite the limitations, all patients undergoing hemorrhoidectomy should consider treatment with PNB.

## Data availability statement

Publicly available datasets were analyzed in this study. This data can be found here: https://pubmed.ncbi.nlm.nih.gov/.

## Author contributions

SX: Conceptualization, Data curation, Formal analysis, Investigation, Methodology, Writing – original draft, Writing – review & editing. LL: Data curation, Formal analysis, Software, Writing – review & editing. WW: Conceptualization, Writing – review & editing, Writing – original draft. KL: Data curation, Software, Writing – review & editing. TJ: Data curation, Software, Writing – review & editing. YL: Data curation, Writing – review & editing.
